# Molecular Targets of Triple-Negative Breast Cancer: Where Do We Stand?

**DOI:** 10.3390/cancers14030482

**Published:** 2022-01-18

**Authors:** Emma E. Newton, Lauren E. Mueller, Scout M. Treadwell, Cindy A. Morris, Heather L. Machado

**Affiliations:** 1 Department of Biochemistry and Molecular Biology, Tulane University School of Medicine, New Orleans, LA 70112, USA; enewton@tulane.edu (E.E.N.); lmueller2@tulane.edu (L.E.M.); streadwell@tulane.edu (S.M.T.); 2 Department of Microbiology and Immunology, Tulane University School of Medicine, New Orleans, LA 70112, USA; cmorris2@tulane.edu; 3 Tulane Cancer Center, Louisiana Cancer Research Consortium, New Orleans, LA 70112, USA

**Keywords:** triple-negative breast cancer, immunotherapy, treatment resistance, biological targets

## Abstract

**Simple Summary:**

Triple-negative breast cancer (TNBC) is one of the most aggressive breast cancer subtypes, largely due to heterogeneity and lack of treatment options. Due to absence of targetable hormone receptor expression, chemotherapy is the current standard of care for TNBC. However, recurrence and metastasis following treatment with chemotherapy and radiation remain major contributors to mortality. To develop more effective treatments of TNBC, molecular pathways involved in tumor growth, vascularization, and apoptosis have been investigated as potential targets. In this review, we outline promising biological targets that may be implicated in future TNBC treatments.

**Abstract:**

Triple-negative breast cancer (TNBC) is a highly aggressive form of breast cancer. Due to its heterogeneity and lack of hormone receptor expression, this subtype is more likely to metastasize and resist treatment attempts than are other forms of breast cancer. Due to the absence of targetable receptors, chemotherapy and breast conserving surgery have been the predominant treatment options for patients. However, resistance to chemotherapy and local recurrence of the tumors is frequent. Emerging immunotherapies have begun to change treatment plans for patients diagnosed with TNBC. In this review, we discuss the various immune pathways identified in TNBC and the role they play as targets for new potential treatment choices. Various therapeutic options that inhibit key pathways in cellular growth cycles, DNA repair mechanisms, epithelial mesenchymal transition, and immunosuppression have been shown to improve survival in patients with this disease. With promising results thus far, continued studies of immunotherapy and neoadjuvant therapy options for TNBC are likely to alter the treatment course for these diagnoses in the future.

## 1. Introduction

Breast cancer is a highly heterogeneous disease comprised of distinct molecular and clinical subtypes that often dictate prognosis, treatment strategy and overall response to therapy. Hormone receptor-positive breast cancers (luminal A and luminal B subtypes) are primarily treated with endocrine therapy that may be combined with other agents that target estrogen signaling. Compared to luminal A, luminal B subtypes typically have lower estrogen receptor and progesterone receptor expression and are associated with an increase in recurrence and poor prognosis [1]. Breast cancers that express human epidermal growth factor receptor 2 (HER2) (luminal B subtype) are initially treated with targeted agents that inhibit HER2 signaling.

However, triple-negative breast cancer (TNBC) is characterized by the lack of expression of estrogen receptor (ER), progesterone receptor (PR) and HER2, rendering targeted therapeutic options limited [2]. Due to its aggressive nature and the lack of effective therapeutic strategies, TNBC is a leading cause of cancer deaths in women [3].

TNBC accounts for 10–15% of all new breast cancer diagnoses [4], with cytotoxic chemotherapy as the mainstay of treatment [5]. Emerging multi-omics approaches have revealed tremendous heterogeneity within TNBC, and thus several different classification schemes have been proposed [6]. The most extensive of these schemes categorizes TNBC into six subtypes: basal-like 1 and 2 (BL1, BL2), immunomodulatory (IM), mesenchymal (M), a mesenchymal stem-like (MSL), and luminal androgen receptor (LAR) subtype [7]. Tumors in the BL1 category modify the cell cycle and cell division pathways in which increased DNA damage responses result in continued proliferation and subsequent tumor progression. Molecular altercations of the BL1 subtype include MYC and RB1 amplifications and more commonly result in invasive ductal carcinoma. Genes altered in the BL2 subtype are associated with growth factor signaling, such as epidermal growth factor, Wnt/β-catenin, and mTOR pathways [8]. The IM subtype mainly proliferates via immune cell and cytokine signaling, and is characterized by alterations in Th1 and Th2 immune responses, as well as natural killer cells. The M subtype is associated with cell motility and cytoskeleton-regulated pathways involving actin. Mutations in cell migration pathways or the epithelial–mesenchymal transition allows for progression as a metastatic malignancy. MSL gene subtypes also function via cell signaling and growth components. Both M and MSL subtypes produce histologically similar sarcoma-like and squamous epithelial cell-like tumors [8]. Finally, the LAR subtype is rather unique in that it is characterized by genes heavily involved in hormone regulation such as steroid synthesis, notably that of androgens and estrogens [6]. The hormone-related LAR subtype is more unique than the other TNBC subtypes, and typically leads to apocrine tumors [8].

The majority of breast cancers are sporadic, but 10–20% of total patients diagnosed with TNBC have a mutation in BRCA1 or BRCA2 [9,10]. BRCA1/2 are tumor suppressor genes that are clinically linked to a hereditary predisposition to developing cancer. Specifically, about 25% of cases have demonstrated BRCA1 mutation, which is typically seen in younger patients [11]. Furthermore, 8% of new TNBC diagnoses are associated with a BRCA2 mutation [2]. Typically, when TNBC is initially diagnosed, the patient will undergo genetic testing to assess whether they have a mutation in BRCA1/2 or are a carrier.

Due to the complex heterogeneity of this disease, TNBC is difficult to treat. However, molecular pathways involved in tumor growth, vascularization, apoptosis, and replication, are now being examined as potential targets for pharmacological intervention. In this review, we outline the tumor markers currently under investigation and ongoing clinical trials and summarize the importance of the tumor microenvironment in the development of these targeted therapies.

## 2. Chemotherapy

Chemotherapy is currently first line for the treatment of TNBC, with or without immunotherapy; as can be seen in Table S1, almost half of current phase III clinical trials involving TNBC focus on chemotherapy. According to current guidelines, women with nonmetastatic tumors larger than 0.5 cm or those who have positive lymph nodes are recommended to receive chemotherapy [12]. Neoadjuvant chemotherapy is used in those for whom the cancer is advanced or are otherwise unlikely to do well with breast conserving surgery. These patients receive a personalized chemotherapy regimen in order to shrink the tumor, and then undergo surgery to remove the remaining mass. This neoadjuvant sequence has been shown to increase the likelihood of pathologic complete response (pCR) [13]. Specifically, anthracycline and taxane-based therapy are associated with 34% pCR [13]. However, a recent meta-analysis confirmed that platinum-based therapy was associated with significant improvement in pCR vs. non-platinum-based therapy (40% vs. 27%, OR = 1.75, 95% CI = 1.36–2.62) [14]. Experts associate platinum-based therapies with more advanced stage cancer unresponsive to anthracycline [15]. For those who have tumors smaller than 0.5 cm, chemotherapy is not typically recommended [16]. For these women, the adverse effects of chemotherapy regimens generally outweigh the risks of the cancer itself. However, research is still being carried out in this area, as the benefits of chemotherapy have still been shown in some of these patients. Therefore, treatment of these women must be individualized based on the diseases’ specific characteristics and other patient factors.

Currently, most patients receive a standard of six to eight cycles of neoadjuvant chemotherapy, although sometimes schedules are limited by toxicity and tumor response. The current adjuvant relapse rate of TNBC is 40% in five years. The current expert consensus is adjuvant anthracycline and taxane-based therapy for six to eight cycles for relapsed patients. For patients unresponsive to the treatment mentioned above, adjuvant capecitabine or platinum-based chemotherapy may be given, although it is controversial. The superiority of either adjuvant capecitabine or platinum-based chemotherapy is unknown, as researchers are currently leading an ongoing randomized phase III trial [15]. Interestingly, each subtype of TNBC responds differently to chemotherapy: BL-1 subtype has shown the greatest response, followed by M subtype, AR subtype, and ending with the BL-2 subtype, which responds poorest to chemotherapy. As recurrence risk following chemotherapy is high, the scientific community is investigating immunotherapy treatments as potential alternatives.

## 3. Tumor Markers and Potential Therapeutic Targets

### 3.1. Immune Checkpoint Inhibitors

The immune checkpoint system is critical for tumor development and is especially important in the progression to metastasis. Programmed cell death protein 1 (PD-1) is a protein expressed on the surface of T-cells. When bound to its ligand, programmed cell death ligand 1 (PD-L1), this complex suppresses T-cell immunity by blocking cytotoxic T-cell activity. Both PD-1 and PD-L1 can be used as markers of T-cell exhaustion and are implicated heavily in tumor progression [17,18]. Cytotoxic T lymphocyte associated protein 4 (CTLA-4) is a similar protein that acts as a receptor on T-cells to downregulate the immune response [17]. CTLA-4 acts early in the T-cell activation process in order to suppress regulatory T-cells. Both CTLA-4 and PD-1/PD-L1 can block tumor infiltrating lymphocytes (TILs) and promote tumor growth and progression. For this reason, drugs that inhibit these complexes, known as immune checkpoint inhibitors (ICIs), are becoming the focus of studies involving multiple types of cancer. In studies involving lung cancer and melanoma, inhibiting both CTLA-1 and PD-1 (with nivolumab and ipilimumab, respectively) has been demonstrated to improve cancer response as compared to chemotherapy alone [19,20]. In one study, median progression-free survival (mPFS) in the group that was treated with nivolumab plus ipilimumab was 7.3 months versus 5.5 months in the group that received only chemotherapy [20]. Interestingly, TNBC has a higher percentage of TILs than many other cancers, and thus is likely to respond better to ICIs [21,22,23,24]. Furthermore, patients with TNBC have been shown to express high levels of PD-L1 [21,25,26], further supporting efforts to inhibit this protein. The abundance of TILs can predict response to chemotherapy and survival rate; if a tumor expresses over 50% TILs, the likelihood of pCR is 40% as compared to 4% in tumors that lack TILs [24]. This effect is even more pronounced if the TILs are CD8+ [27,28].

Pembrolizumab is a monoclonal antibody directed against PD-1 that was originally developed to target metastatic melanoma [18]. However, due to the increasing research on PD-1 in TNBC, several trials have been developed in order to evaluate its efficacy in this disease. Initially, in a phase Ib study of patients with PD-L1-positive TNBC, pembrolizumab demonstrated an overall response rate (ORR) of 18.5% [29]. Since then, multiple studies have been conducted using pembrolizumab monotherapy in combination with chemotherapy. Studies of monotherapy with pembrolizumab in metastatic disease have been largely unsuccessful, but this drug in combination with chemotherapy has been shown to be effective. KEYNOTE-355, a phase III trial, demonstrated improved mPFS using pembrolizumab in combination with standard chemotherapy (9.7 months) compared to chemotherapy with a placebo in TNBC patients whose tumors expressed PD-L1 (5.6 months) [30]. Subsequently, the KEYNOTE-119 study showed that in PD-L1-positive patients with metastatic TNBC, median overall survival (OS) increased from 11.6 months in the group that received chemotherapy alone to 12.7 months in the pembrolizumab/chemotherapy combination group. However, the effects were insignificant in those whose tumors did not express PD-L1 [31]. In another study examining the pCR of pembrolizumab combined with chemotherapy versus chemotherapy alone, the pCR rate in the combination group was three times that of chemotherapy alone in patients with stage II/III TNBC [29]. Pembrolizumab is now FDA approved for the treatment of metastatic TNBC in PD-L1+ patients [16].

Another drug developed to interfere with the immune checkpoints is atezolizumab, a humanized IgG1 antibody targeting PD-L1. Atezolizumab enhances T-cell suppression by blocking the interaction of PD-L1 with PD-1 and B7-1, which is another protein on antigen-presenting cells that inhibits T-cell suppression [32]. An ORR of 10% was demonstrated in a phase 1 trial [33], but in a subsequent phase Ib trial, monotherapy showed an ORR of only 5.2% [34]. More promising results were obtained in studies that combined atezolizumab with chemotherapy. A 1.7-month increase in PFS (7.2 months versus 5.5 months, respectively) was demonstrated when combining atezolizumab with chemotherapy compared to chemotherapy with placebo [32]. This effect was improved in the PD-L1-positive subgroup, which showed a 7.5-month PFS as compared to 5.0 months in the placebo group. This PD-L1 group also demonstrated a 25-month overall survival when given the combination of atezolizumab and chemotherapy, compared to 18 months in the chemotherapy and placebo group [32]. Atezolizumab in combination with nab-paclitaxel is now FDA approved for treatment of TNBC patients with PD-L1+ cancer [16]. Due to the successes of these studies, focus is now shifting to combinations of atezolizumab with different forms of chemotherapy. Impassion031, a phase III clinical trial, examined atezolizumab combined with nab-paclitaxel followed by doxorubicin plus cyclophosphamide in patients with early stage TNBC. In PD-L1-positive patients, pCR was reached in 69% of atezolizumab patients versus 49% in the control group that received chemotherapy with placebo [35]. Unfortunately, studies examining the effects of atezolizumab combined with chemotherapy in early stage TNBC have not been as promising as those conducted on pembrolizumab as discussed above [36]. Furthermore, when combined with other therapeutic targets such as MEK, PD-1 inhibition was shown to significantly decrease the size of murine syngeneic tumor models [37].

As shown in Table 1, the vast majority of current clinical trials involving immunotherapy treatments of TNBC are focused on immune checkpoint inhibitors. As detailed above, many of these trials have shown promising, statistically significant increases in PFS and pCR. However, it must be noted that in most of these trials, the interventions are increasing survival by mere months, and that these drugs are by no means a cure to this disease. More research must be conducted to further elucidate the role of immune checkpoint inhibitors in the treatment of TNBC, especially in those with PD-L1-positive tumors.

### 3.2. Antibody Drug Conjugates

Recent studies have examined antibody drug conjugates (ADCs), which are monoclonal antibodies that target cancer cells and administer high-dose cytotoxic drugs directly to their intended destinations. Anti-trophoblast cell surface antigen 2 (Trop2) is an ADC that is commonly overexpressed in cancer cells [38], and is active in the MAPK-ERK1/2 pathways [39,40]. Trodelvy (Sacituzumab-govitecan), an anti-Trop2 humanized monoclonal antibody, demonstrated an increased PFS rate of 5.5 months in TNBC patients compared to 1.7 months with standard chemotherapy alone [41]. In patients with metastatic TNBC, Sacituzumab-govitecan is FDA approved for those who have failed two prior therapies [16]. Another ADC, trastuzumab-deruxtecan, targets HER2 and has been shown to benefit patients with HER2-positive breast cancer. This combination is now the subject of several clinical trials involving metastatic TNBC [42].

### 3.3. Receptor Tyrosine Kinase Pathways

Receptor tyrosine kinases (RTKs) are transmembrane receptors that are activated by extracellular ligands to launch signaling cascades that mediate numerous cell functions. In TNBC, numerous RTKs are overexpressed including EGFR, FGFR1, PDGFR, ERBB3, ERRB4 and Axl [40], all of which dimerize to induce downstream pathways that mediate growth, apoptosis and survival [43]. For example, RTK-induced Ras GTPase leads to activation of Raf. When Raf is activated, it phosphorylates MEK1 and MEK2, which are dual-specificity tyrosine and serine/threonine kinases. These then activate ERK1/2, which have several downstream effects relevant to TNBC including cancer cell proliferation. RTKs can also activate PI3K-dependent AKT-mTOR signaling to lead to the transcription of genes important for cancer cell survival [44].

While RTK activation is dependent on growth factor signals in normal physiology, cancer cells adopt mechanisms to ensure constitutive activation and ligand-independent RTK signaling, leading to uncontrolled proliferation. Thus, targeting RTK signaling has long been an attractive therapeutic strategy. In TNBC cell lines, an MEK inhibitor called Selumetinib has been shown to reduce migration and proliferation. This drug also prevented lung metastases in mouse xenograft models [45]. Expression of another molecule in this pathway, ERK, has been shown to be negatively associated with the 5-year OS rate in TNBC patients. Among women with TNBC, those whose tumors overexpressed ERK-2 had a 5-year OS of 57%, versus 79% in those with low ERK-2 levels [46]. Drugs targeting AKT have been studied in this pathway as well: Ipatasertib, an AKT inhibitor, has been investigated as a first-line drug in TNBC patients. When combined with paclitaxel, median PFS increased from 4.9 in the chemotherapy alone subgroup to 6.2 months in those who received the combination of chemotherapy with ipatasertib [47]. PI3K is another potential target, as activating mutations in this molecule are seen in 23.7% of TNBC patients [48]. Inhibition of this molecule has been shown to cause increased sensitivity to poly(ADP-ribose) polymerase (PARP) inhibition, which may allow for PARP inhibitors to be used successfully [48].

Axl is a member of the TAM (Tyro3, Axl, Mer) receptor family that is normally expressed on macrophages and regulates the clearance of apoptotic cells during the innate immune response to infection. Axl is overexpressed in a number of cancers, including TNBC [49], and has been shown to promote proliferation, survival and invasion of cancer cells [50,51]. In non-small cell lung cancer, Axl was shown to have a central role in overcoming resistance to therapies that target EGFR [52].Thus, a number of studies have aimed to target Axl as a therapeutic strategy. Growth arrest specific gene 6 (Gas6) is a ligand of Axl, and studies are also examining Gas6/Axl signaling in order to further explore this pathway. In an ex vivo study, it was shown that the induction of premalignant cells by macrophages was dependent on Gas6 signaling [53]. Interestingly, an analysis of breast cancer patients with high Gas6 mRNA expression showed improved OS and relapse free survival, suggesting that the interaction is more complex than a simple linear relationship [54]. Since these pathways can be pharmacologically targeted in numerous ways, including anti-Axl monoclonal antibodies, small molecule inhibitors, soluble receptors, nucleotide aptamers and natural compounds, it is no surprise that there are over 30 current clinical trials that are targeting Axl and its effects on multiple different cancer types [55]. Specifically examining the role of Axl in TNBC, in a study conducted in 2016, treatment with an anti-AXL monoclonal antibody inhibited in vitro metastasis of AXL-positive cancer cells [49]. A phase II trial of bemcentinib, an oral AXL inhibitor, in combination with pembrolizumab in patients with TNBC and adenocarcinoma of the lung, has completed data collection and results are pending [56].

Crosstalk between RAS/MAPK and PI3K AKT/mTOR pathways has been shown to promote treatment-resistant growth in many cancers. It is possible that drugs targeting the communication between these pathways could be an effective method of treatment. EGFR, a communicating molecule, is overexpressed in 60–80% of TNBC tumors, and other molecules such as PDGFR, VEGF, IGFR, C-Met and TGFR have been implicated as well [57]. Bevacizumab, an anti-VEGF monoclonal antibody, showed a clinical response rate (CRR) of 96% when combined with docetaxel carboplatin in a phase 2 clinical trial [58]. In a network meta-analysis, regimens containing bevacizumab maintained a higher pCR than the standard regimens to which they were compared [59]. An in vitro study examined the effects of dual blockade of EGFR and mTOR in CAL-51 cells, a PI3K and PTEN-mutant cell line. Gefitinib and Everolimus were used to inhibit EGFR and mTOR, respectively, and this combination significantly increased apoptosis, downregulated several cell cycle regulators and subsequently slowed progression of the cell cycle [60]. Although these dual and triple therapies are showing promise in terms of slowing tumor progression and reducing tumor burden, it must also be noted that this method of treatment is particularly toxic and may likely be difficult to properly dose if ever approved.

### 3.4. Androgen Receptor and Coordinating Pathways

Although TNBC lacks ER and PR, many patients have been shown to be androgen receptor (AR)-positive. Identifying and targeting this pathway provides a hormonal treatment option for a TNBC, which typically resists this type of therapy. AR is commonly targeted in prostate cancer, where anti-androgens are relatively effective. Biclutamide, a well-known AR inhibitor often used to treat AR+ prostate cancer, was shown to increase PFS in AR+ TNBC in a phase II clinical trial [61]. A similar drug, enzalutamide, showed clinically significant results and was well tolerated by patients in a phase II study [62]. Similar to the aforementioned multi-drug regimens targeting cross talk, AR receptor antagonists can be combined with PI3K inhibitors. In a phase II trial of TNBC patients, the median PFS was 2.7 months in those who received a combination of enzalutamide and taselisib (a PI3K inhibitor). The clinical benefit rate at 16 weeks was also significantly higher in this combination group clinical benefit rate in patients who received the combination therapy [63].

### 3.5. Wnt/β-Catenin

The Wnt/β-catenin pathway is a protein set that is involved in many processes relevant to tumor development, including tumor recurrence, stem cell regeneration and cellular repair [64]. The Wnt/β-catenin pathway is upregulated in TNBC, and activation of Wnt is associated with poor prognosis in patients with this disease [65]. When Wnt forms a complex with two proteins called Frizzled and LRP5/6, phosphorylated LRP5/6 recruits axin, a scaffold protein, to stabilize β-catenin. These molecules work together to regulate tumor cell proliferation and differentiation, leading to increased disease progression. Due to the inverse association between Wnt expression and prognosis of patients with TNBC, there are several clinical trials currently targeting this pathway. Niclosamide, an anti-helminthic drug classically used to treat tapeworm infections, has been shown to reduce LRP6 in both in vitro and in vivo TNBC xenografts [66]. Clofazimine, a common anti-mycobacterial used to treat leprosy, has been shown to inhibit Wnt signaling in in vitro TNBC cells [67]. Finally, suramin, a drug that was originally developed to treat African Sleeping Sickness, has been shown in vitro and in mouse xenografts to inhibit the proliferation of TNBC cells via inhibition of Wnt [68].

### 3.6. DNA Damage Repair

Typically, cell repair mechanisms ensure that cells with damaged DNA undergo either repair or apoptosis. Therefore, inhibition of these mechanisms can lead to a buildup of damaged DNA in cells resulting in apoptosis or senescence of the tumor. Due to the frequent cell turnover and high mutation rate in TNBC, PARP inhibitors have been closely examined as a treatment mechanism for patients with this cancer. Poly adenosine diphosphate-ribose polymerase (PARP) is an enzyme that repairs damaged DNA in cells. In TNBC, this molecule can be targeted to prevent cancer cells from repairing themselves. Two PARP inhibitors, olaparib and talazoparib, are FDA approved for TNBC patients with BRCA mutations who have shown resistance to chemotherapy [16], and there are several other current clinical trials examining the effectivity of PARP inhibitors on TNBC. Talazoparib has also shown promising clinical effectivity as neoadjuvant therapy. In a small study in which patients were treated with talazoparib before undergoing surgery, 53% of patients achieved pCR [69]. Talazoparib has also shown efficacy in in vitro TNBC cell lines and is currently the subject of a phase II clinical trial as maintenance therapy for TNBC [70]. Iniparib, another PARP inhibitor, in combination with chemotherapy has been shown in a phase II clinical trial to have a 43–56% clinical benefit rate and a 32–52% ORR [71]. PARP inhibitors have recently also been shown to upregulate PD-L1 expression in animal models [72,73], which may lead to future studies focusing on combinations involving the various PD-L1 drugs discussed above.

PARP inhibitors have been shown to be more effective in patients with BRCA1/2 mutations, likely due to their involvement in DNA damage repair [74]. In a study comparing olaparib to single-agent chemotherapy in TNBC patients with a germline BRCA mutation, those who received olaparib had an ORR of 59.9% versus 28.8% in the control group who received chemotherapy alone [75]. Furthermore, olaparib and talazoparib have been shown to improve PFS compared to chemotherapy alone in three separate randomized clinical trials in patients with BRCA1/2 mutations [76]. Although the treatment potential for PARP inhibitors in wild-type BRCA patients is unknown, both preclinical and in vivo studies are showing promise for their efficacy in breast cancers without BRCA1/2 mutations [77,78,79,80]. Additionally, some cancers that do not have actual mutations in BRCA1/2 have high levels of a quality known as “BRCAness”, which indicates their similarity to cancers with mutations in these genes. Current research is investigating the potential that PARP inhibitors may work in cancers with wild-type BRCA1/2 but high levels of BRCAness [81]. The basal-like subtype of TNBC has been shown to demonstrate high levels of this quality, implying that PARP inhibitors may be effective for this patient group [81].

### 3.7. EMT and Associated Pathways

Triple-negative breast cancer is known for its treatment resistance and proclivity for metastasis. Because it is so aggressive, treatments involved with inhibiting a transition that allows for further metastasis is critical to prevent advanced disease. The epithelial mesenchymal transition (EMT) is implicated in the transition from a localized tumor to a metastatic one, as cells need to undergo this transition in order to mobilize and travel to distant sites. There are several genes that have been implicated in EMT, and altered expression of micro-RNA has been shown to contribute to EMT in TNBC. Specifically, overexpression of MiR-200b-3p and miR 5p has been demonstrated to suppress migration of TNBC cells and their subsequent transition into a mesenchymal phenotype [82].

The transition to a mesenchymal phenotype has also been shown to be reversed by histone deacetylase inhibitors. Panobinostat, a pan-deacetylase inhibitor, has been shown to increase histone acetylation, decrease tumor cell proliferation, and block cell cycle progression in four TNBC cell lines in vitro. The same study also showed an increase in induction of apoptosis in three cell lines treated with panobinostat [83]. This drug has also been shown to reverse EMT in vivo and inhibit metastasis [84]. In one study, panobinostat significantly inhibited TNBC gene expression both in vitro and in vivo, indicating a strong therapeutic potential for future investigation [84].

### 3.8. Protein Stability Agents

Protein stability inducing agents, such as the heat shock protein 90 (HSP90), are also implicated in the progression and metastasis of TNBC [85]. HSP90 is a molecular chaperone responsible for the stability and activation of over 200 proteins involved in cellular replication, signaling, and growth [85]. In a preclinical study, two HSP90 inhibitors known as celastrol and triptolide were shown to suppress proliferation and clonogenicity of cells in four TNBC cell lines; Western blots of the results showed reduced levels of BRCA1 in the nucleus [86]. In clinical trials, a HSP90 inhibitor called Ganetespib led to downregulation of several proteins important to TNBC such as EGFR, IGF-1R, MET and CRAF [87]. HSP90 inhibitors show promise in treating TNBC in clinical trials, but not all patients respond consistently to therapy [88,89].

## 4. Tumor Markers and Potential Targets

TNBC is characterized by a unique tumor microenvironment (TME) that allows for cancer cells to grow and metastasize efficiently. Tumor-associated macrophages (TAMs) are abundant in TNBC and are associated with poor prognosis [90,91,92]. Numerous subsets of TAMs have been described, which have diverse tumor-promoting functions, including the mediation of chemoresistance, T-cell suppression and promotion of angiogenesis [93,94,95]. Myeloid-derived suppressor cells (MDSCs) are neutrophils that can invade tumors and suppress T-cell proliferation [96]. MDSCs in the TME secrete immunosuppressive factors such as TGFβ and IL-10, which transform TAMs into a more aggressive phenotype [97]. These can secrete reactive oxygen species that limit T-cell proliferation by depleting the TME of nutritive molecules [98]. MDSCs also promote tumor cell metastasis by aiding in angiogenesis and inducing the epithelial–mesenchymal transition [99]. An important prognostic marker associated with the TME is the neutrophil–lymphocyte ratio. As mentioned above, neutrophils encourage angiogenesis, which allows localized tumors to metastasize to distant sites. In TNBC patients, tumors with a high neutrophil–lymphocyte ratio (NLR) have been shown to be significantly more likely to achieve pathologic complete response [100,101,102]. Furthermore, an inverse relationship has been shown between high NLRs and PFS and overall survival rates [100].

Multiple studies targeting TAMs and MDSCs have been developed in order to halt tumor progression via the aforementioned mechanisms. In vivo experiments using murine models demonstrate that stromal TAM depletion with CSF1R, a TAM differentiation/recruitment factor [103], can increase both the number of T-cells present in tumor islets and their motility [104]. Direct inhibition of this interaction between CSF-1 and CSF-1R, in combination with paclitaxel, was shown to inhibit TAMs and improve infiltration of T-cells into tumor islets [103]. Mer, a phagocytic RTK expressed by macrophages, allows tumors to grow by clearing tumor cell debris. Inhibition of this receptor allows apoptotic bodies in the TME to accumulate and halt tumor progression.

## 5. Resistance to Treatment

Resistance to chemotherapy, checkpoint inhibitor treatment, and other biological agents are implicated in the low success rate in treatment of TNBC. Resistance is multifactorial and requires a combination of therapies and close clinical oversight to overcome. Tumor resistance is conferred by avoidance of apoptosis, increased cellular proliferation, and immune evasion.

Regarding neoadjuvant chemotherapy, half of TNBC patients develop chemoresistance to treatment, contributing to the associated low survival rate of TNBC [12]. A class of ABC transporters have been implicated in conferring resistance to chemotherapy treatment and downregulation of these transporters has mitigated resistance to chemotherapy treatments [105,106]. An increase in the presence of cancer stem cells is also implicated in TNBC resistance, and is associated as an indicator of poor prognosis [107]. Researchers have established that the deregulation of the Wnt pathway is associated with tumor proliferation and metastasis due to treatment resistance [108]. The mechanism of resistance is stabilization of β-catenin by stabilization of the Wnt ligand receptor, blockage of Wnt ligand antagonists, or loss of APC tumor suppressor [109,110]. The tumor microenvironment may also confer resistance by reducing T-cell infiltration, as TGFBR2 in CD4+ T-cells has been implicated in inducing cancer cell hypoxia [111,112]. Finally, somatic mutations in BRCA1 due to cytotoxicity may inhibit effectiveness of PARP inhibitors in TNBC, conferring specific resistance to this treatment [113,114]. To avoid tumor resistance and further metastasis, combination therapy is the current standard of care.

## 6. Conclusions

**Figure 1:**
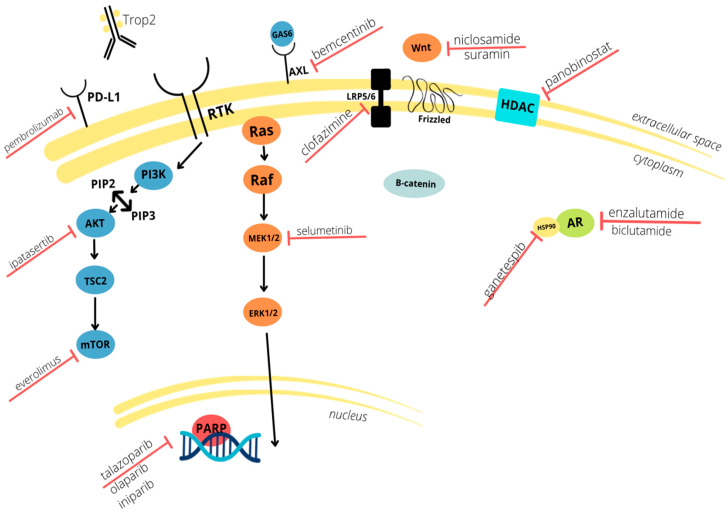
Schematic depiction of the molecular mechanisms involved in TNBC (Triple-Negative Breast Cancer) pathogenesis and the biomarkers targeted by neoadjuvant therapies discussed in this review. Current clinical trials focus on both intra and extracellular pathways.

TNBC is an extremely aggressive and difficult to treat subset of breast cancer. Due to the lack of ER, PR, and HER2 receptors, targeted hormone therapies are not an option, and thus chemotherapy is the mainstay of treatment. Though there are five immunotherapy drugs that are FDA approved for the treatment of TNBC, chemotherapy (followed by surgical intervention for larger tumors) is still the mainstay of treatment. In an effort to improve treatment outcomes, immunotherapy drugs targeting EGFR, VEGF, and PARP pathways have been studied to improve treatment options. Other molecules known to play a role in the epithelial mesenchymal transition are also being investigated, as well as those involved in immune system checkpoints and DNA repair. These tumor markers and the drugs with which they interact are summarized in Figure 1 below. With continued studies, newly developed immunotherapeutics may prove to become the first-line treatments for primary diagnoses of TNBC and may also potentiate increased survival of patients with metastasis or recurrence.

## Figures and Tables

**Table 1:** Current Immunotherapy Clinical Trials.

Drug Type	Drug	Total Trials	Completed	Active
**Immune checkpoint** **inhibitors**	pembrolizumab	90	11	79
	atezolizumab	55	3	52
**Antibody-drug** **conjugates**	sacitizumab-govitecan	7	1	6
	trastuzumab-deruxtecam	2	0	2
**RTK pathway** **inhibitors**	selumetinib	2	0	2
	ipatasertib	9	2	7
	bemcentinib	1	1	0
	bevacizumab	26	13	13
**EMT pathway** **inhibitors**	panobinostat	3	1	2
**Androgen receptor** **inhibitors**	bicalutamide	4	0	4
	enzalutamide	8	1	7
	taselisib and enzalutamide	1	1	0
**DNA damage** **repair inhibitors**	iniparib	7	6	1
	olaparib	26	7	19
	talazoparib	13	0	13

RTK—receptor tyrosine kinases, EMT—epithelial mesenchymal transition.

## Supplementary Materials

The following supporting information can be downloaded at: https://www.mdpi.com/article/10.3390/cancers14030482/s1, Table S1: Current phase III clinical trials involving triple negative breast cancer.
