# Supplementary material for: Molecular Targets of Triple-Negative Breast Cancer: Where Do We Stand?
Source: Cancers (Basel). 2022 Jan 18;14(3):482. doi: 10.3390/cancers14030482 (PMC8833442; doi:10.3390/cancers14030482)
Supplement: Supplementary file 1 [file cancers-14-00482-s001.zip › cancers-1527199-supplementary.pdf]

Table S1: Current Phase III Clinical Trials Involving Triple Negative Breast Cancer

Category	Identifier	Status	Main Intervention
**Immune Checkpoint Inhibitors**	NCT04301739	Not yet recruiting	Serplulimab
	NCT04177108	Active; not recruiting	Atezolizumab, ipasertib
	NCT04613674	Recruiting	Camrelizumab
	NCT04148911	Recruiting	Atezolizumab
	NCT04085276	Recruiting	Toripalimab
	NCT03498716	Recruiting	Atezolizumab
	NCT03281954	Active; not recruiting	Atezolizumab
	NCT03197935	Active; not recruiting	Atezolizumab
	NCT03125902	Active; not recruiting	Atezolizumab
	NCT02425891	Completed	Atezolizumab
	NCT02555657	Completed	Pembrolizumab
	NCT02819518	Active; not recruiting	Pembrolizumab
	NCT04335006	Recruiting	Camrelizumab, apatinib
	NCT03371017	Recruiting	Atezolizumab
	NCT03036488	Active; not recruiting	Pembrolizumab
	NCT02926196	Active; not recruiting	Avelumab
	NCT03777579	Suspended	Toripalimab
	NCT04907344	Not yet recruiting	Camrelizumab
	NCT04191135	Active; not recruiting	Pembrolizumab, olaparib
	NCT04799249	Recruiting	Trilaciclib
	NCT02620280	Active; not recruiting	Atezolizumab

**Antibody-Drug Conjugates**	NCT02574455	Completed	Sacitizumab govitecan
	NCT04595565	Recruiting	Sacitizumab govitecan
**RTK Pathway Inhibitors**	NCT04405505	Not yet recruiting	Anlotinib
	NCT04177108	Active; not recruiting	Ipasertib, atezolizumab
	NCT04335006	Recruiting	Apatinib, camrelizumab
	NCT03337724	Active; not recruiting	Ipatasertib
	NCT03997123	Recruiting	Capivasertib
	NCT04251533	Recruiting	Alpelisib
	NCT04405505	Not yet recruiting	Anlotinib
**Androgren Receptor Inhibitors**	NCT03055312	Terminated	biclutamide
	NCT02929576	Withdrawn	enzalutamide
**DNA Damage Repair Inhibitors**	NCT02032277	Completed	Veliparib
	NCT04915755	Recruiting	Niraparib
	NCT03150576	Recruiting	Olaparib
	NCT00938652	Completed	Iniparib
**Antibiotics**	NCT04722978	Recruiting	Moxifloxacin
**Bisphosphonates**	NCT02595138	Active; not recruiting	Zoledronic acid
**Herbal**	NCT04403529	Recruiting	Traditional chinese medicine formation (12 herbal components)
**Vaccine**	NCT03562637	Recruiting	Adagaloxad simolenin

**L-Aspariginase**	NCT03674242	Recruiting	Eryaspase
**VEGF Inhibition**	NCT00528567	Completed	Bevacizumab
**Liposomal Targeting**	NCT03002103	Recruiting	EndoTAG-1
**Chemotherapy**	NCT01216111	Recruiting	Chemotherapy
	NCT01216111	Completed	Chemotherapy
	NCT03168880	Active; not recruiting	Chemotherapy
	NCT04296175	Recruiting	Chemotherapy
	NCT03876886	Recruiting	Chemotherapy
	NCT02546934	Active; not recruiting	Chemotherapy
	NCT01287624	Completed	Chemotherapy
	NCT04137653	Recruiting	Chemotherapy
	NCT01642771	Unknown	Chemotherapy
	NCT04031703	Completed	Chemotherapy
	NCT02441933	Recruiting	Chemotherapy
	NCT02455141	Recruiting	Chemotherapy
	NCT02641847	Unknown	Chemotherapy
	NCT01150513	Completed	Chemotherapy
	NCT04335669	Recruiting	Chemotherapy
	NCT01112826	Completed	Chemotherapy
	NCT00532727	Unknown	Chemotherapy
	NCT01881230	Completed	Chemotherapy

	NCT02488967	Recruiting	Chemotherapy
	NCT00789581	Completed	Chemotherapy
	NCT02225470	Completed	Chemotherapy
	NCT01057069	Active; not recruiting	Chemotherapy
	NCT01805076	Active; not recruiting	Chemotherapy
	NCT00130533	Completed	Chemotherapy
	NCT02445391	Recruiting	Chemotherapy
	NCT00912444	Terminated	Chemotherapy
	NCT00630032	Completed	Chemotherapy
	NCT02207335	Unknown	Chemotherapy
	NCT01662128	Recruiting	Chemotherapy
	NCT00022516	Completed	Chemotherapy
